# The association between bone turnover biomarkers and the severity of white matter hyperintensities

**DOI:** 10.3389/fneur.2025.1624505

**Published:** 2025-09-19

**Authors:** Qian You, Ying Li, Yufeng Ge, Xiaofan You, Xufeng Chen, Lin Lei, Hongtao Hu

**Affiliations:** ^1^Department of Neurology, Beijing Jishuitan Hospital, Capital Medical University, Beijing, China; ^2^Department of Orthopaedics and Traumatology, Beijing Jishuitan Hospital, Capital Medical University, Beijing, China

**Keywords:** bone turnover biomarkers, white matter hyperintensities, parathyroid hormone, carboxy-terminal cross-linked telopeptide of type 1 collagen, cerebral small vessel disease

## Abstract

**Background:**

Bone health may be associated with cerebral small vessel disease. This study aims to explore the correlation between bone turnover biomarkers (BTMs) and parathyroid hormone (PTH) with the severity of white matter hyperintensities (WMH).

**Materials and methods:**

We retrospectively analyzed 213 inpatients from the Neurology Department of Beijing Jishuitan Hospital between June 2021 and May 2022. The WMH burden was assessed semi-quantitatively using the age-related white matter changes scale and the Fazekas scale, with the latter separately evaluating periventricular WMH (PWMH) and deep WMH (DWMH). Participants were categorized into two groups based on WMH severity. Binary logistic regression was performed to investigate the relationship, and subgroup analyses were conducted across subgroups stratified by sex, hypertension, and diabetes.

**Results:**

Patients with severe WMH, PWMH, and DWMH had significantly higher β-carboxy-terminal cross-linked telopeptide of type 1 collagen (β-CTX) and PTH levels compared to those in the mild groups. After adjusting for confounding factors, elevated β-CTX levels remained associated with severe WMH (OR: 4.44, 95% CI: 1.33–14.81) and severe PWMH (OR: 6.37, 95% CI: 1.80–22.47), while increased PTH levels were associated with severe DWMH (OR: 1.02, 95% CI: 1.01–1.05). Subgroup analysis showed a significant relationship between PTH and the severity of WMH and PWMH in patients with diabetes (*p* for interaction < 0.05).

**Conclusion:**

β-CTX was independently linked to WMH/PWMH severity, and PTH to DWMH severity, pointing to a possible bone–cerebrovascular axis. Larger prospective and interventional studies should confirm these markers as potential CSVD biomarkers and treatment targets.

## 1 Introduction

Cerebral small vessel disease (CSVD) is a leading cause of stroke, vascular dementia, and vascular parkinsonism. It is also associated with gait abnormalities, recurrent falls and mood disorders, significantly impairing brain health and imposing heavy burden on families and society (1). White matter hyperintensities (WMH), a characteristic imaging feature of CSVD, are highly prevalent. In low-income and middle-income countries, the median prevalence of moderate-to-severe WMH is reported to be 20.5% in the general community, 40.5% in stroke patients, and 58.4% in individuals with dementia (2). In high-income countries, the prevalence of WMH among elder adults is relatively high, ranging from 65 to 96% (3). WMH are independently and significantly associated with stroke, mild cognitive impairment and dementia (3, 4). WMH start slowly and progress gradually, with no early biomarkers available for screening or prediction. Because the exact cause is still unclear, treatment options are limited. Therefore, further research on WMH is highly needed.

In recent years, growing interest in cross-organ crosstalk has brought the emerging concept of the “bone–brain axis” into focus. Bone is now recognized as an atypical endocrine organ that contributes to systemic energy and mineral homeostasis (5). Growing evidence links abnormal bone metabolism to brain dysfunction in neurodegenerative diseases, though the mechanisms are still unclear (6, 7). The bone–brain axis explores how bone metabolism affects the brain, aiming to find new ways to prevent and treat neurological diseases.

Bone tissue undergoes continuous remodeling and reconstruction through the dynamic process of bone turnover, which is mediated by osteoclasts and osteoblasts. Disruption of bone turnover is a key pathophysiological mechanism underlying various bone diseases. Bone turnover biomarkers (BTMs) are generated as metabolic byproducts during bone turnover. These biomarkers reflect the levels and activities of bone formation and resorption, making them valuable for the diagnosis and differentiation of bone disease. BTMs are typically categorized into markers of bone formation and resorption. Procollagen type 1 N-terminal propeptide (P1NP), a product of type 1 procollagen cleavage, is a sensitive indicator of systemic bone formation, while osteocalcin (OC), the most abundant non-collagen protein in bone tissue, also reflects bone formation activity and is involved in regulating glucose and lipid metabolism. On the other hand, carboxy-terminal cross-linked telopeptide of type 1 collagen (CTX), a degradation product of type 1 collagen, serves as a highly sensitive and specific marker of bone resorption, with β-CTX being particularly reliable (8). Bone resorption and formation are regulated by endocrine hormones, with parathyroid hormone (PTH) playing an important role.

Osteoporosis and atherosclerosis frequently coexist—both are age-related public-health challenges that share many risk factors (9). Recently, the possible connection between bone health and vascular diseases has attracted growing attention. Studies have shown that bone mineral density (BMD) loss is significantly associated with an increased burden of CSVD (10). Circulating bone-resorption markers are higher in individuals with increased carotid intima-media thickness and correlate positively with brachial–ankle pulse-wave velocity (11). Studies have shown osteoporosis and atherosclerosis may intersect through similar molecular pathways—bone and vascular mineralization, lipid-oxidation products, and chronic inflammation (9). PTH, a key endocrine regulator of bone turnover, also has systemic vascular effects. Elevated PTH levels have been reported in patients with ischemic stroke and are associated with greater carotid intima-media thickness and more severe WMH (12–14). In patients with primary hyperparathyroidism and concurrent hypertension, parathyroidectomy often lowers blood pressure, possibly because excess PTH stimulates renin release and calcium–phosphate imbalance impairs endothelial relaxation (15, 16). Together, these observations imply that PTH might promote CSVD by disrupting endothelial homeostasis, promoting calcium–phosphate dysregulation, and aggravating hypertension.

These findings suggest that bone-related metabolic factors may influence the development and severity of WMH. Despite growing evidence, studies linking bone metabolism to WMH remain limited and unsystematic. Thus we examined two formation markers (P1NP and OC), one resorption marker (β-CTX), and the regulatory hormone PTH. By testing their associations with WMH, we aim to explore potential biological links between altered bone metabolism and CSVD and to offer a practical hypothesis for future mechanistic and clinical work.

## 2 Materials and methods

### 2.1 Ethics approval

Ethical approval for this study was obtained from the Ethics Committee of Beijing Jishuitan Hospital (Approval No. Ji Lun [K2025] No. 164-00). Informed consent was waived due to the retrospective design and minimal use of personal information.

### 2.2 Participants

We retrospectively reviewed the medical records of inpatients from the Neurology Department of Beijing Jishuitan Hospital between June 2021 and May 2022. Adult patients who had undergone both brain magnetic resonance imaging (MRI) and BTM testing were included in the study. Patients were excluded if they met any of the following criteria: (1) A medical history affecting BTMs (such as multiple myeloma, bone tumor, recent fractures within the past 3 months and parathyroid dysfunction). (2) Use of medications known to influence BTMs (such as corticosteroids, heparin, warfarin and antiepileptic drugs). (3) White matter lesions caused by other causes (such as central nervous system demyelinating diseases and progressive multifocal leukoencephalopathy). (4) Difficulty in evaluating WMH (such as extensive cerebral infarction). (5) Pregnancy or postpartum within 1 year.

### 2.3 Data collection

Patient data were extracted from medical records, including demographic details (age and sex) and clinical information such as history of hypertension, diabetes mellitus (DM), cerebral infarction, smoking, height and weight. Hypertension was defined as a systolic blood pressure ≥140 mmHg, diastolic blood pressure ≥90 mmHg, or the use of antihypertensive medication. DM was defined as hemoglobin A1c > 6.5%, self-reported diabetes, or the use of antidiabetic medication. Body mass index (BMI) was calculated as weight (kg) divided by height squared (m^2^), and obesity was defined as BMI ≥ 28 kg/m^2^ (17). Laboratory test results were collected, including levels of total cholesterol (TCHO), triglycerides (TG), high-density lipoprotein (HDL), low-density lipoprotein (LDL), homocysteine (HCY), serum creatinine, uric acid (UA), serum calcium, serum phosphate, total procollagen type 1N-terminal propeptide (tP1NP), β-CTX, OC, PTH and 25-hydroxyvitamin D_3_ (25[OH]D_3_). Albumin-corrected calcium was calculated using a modified formula: total serum calcium (mmol/l) + [40-albumin (g/l)] × 0.018 (18). BTMs were measured using fasting morning blood samples collected after hospital admission to minimize the influence of diet and physical activity on the results.

### 2.4 WMH evaluation

WMH were evaluated using brain MRI performed on 3.0T (Philips Healthcare, Best, The Netherlands) or 1.5T scanners (GE Healthcare, Milwaukee, WI, United States), including at least four sequences: T1-weighted imaging, T2-weighted imaging, fluid-attenuated inversion recovery (FLAIR) and diffusion-weighted imaging (DWI). The FLAIR sequence was primarily used for the semi-quantitative assessment of WMH. The burden of WMH was assessed using the age-related white matter changes (ARWMC) scale and the Fazekas scale, with the latter separately evaluating periventricular WMH (PWMH) and deep WMH (DWMH). Participants were categorized into two groups based on WMH severity evaluated by different classification separately: the mild group (ARWMC score ≤10, PWMH Fazekas score 0–1, DWMH Fazekas score 0–1) and the severe group (ARWMC score >10, PWMH Fazekas score 2–3, DWMH Fazekas score 2–3). The WMH scoring was independently performed by two raters (Qian You and Ying Li), and discrepancies were resolved by consultation with a third expert (Hongtao Hu). The raters were blinded to clinical and laboratory information.

### 2.5 Data analysis

Data analysis was performed using R 4.3.0. Descriptive statistics were used to summarize baseline characteristics: categorical variables were presented as percentages, and continuous variables were expressed as mean ± standard deviation (for normally distributed data) or median and interquartile range (for non-normally distributed data). Univariate analysis was conducted to compare variables between groups. Independent sample t-tests were used for continuous variables with a normal distribution, while the Mann–Whitney *U* test for non-normally distributed continuous variables. Chi-square tests were used for categorical variables. Based on the results of univariate analysis, variables with potential associations (*p* < 0.1) were included in binary logistic regression models. Subsequently, adjustments were made for potential confounders, including age, sex, BMI, hypertension, DM, history of stroke, serum creatinine, corrected calcium levels and phosphate levels. Finally, subgroup analyses were performed based on sex, hypertension and diabetes status. Forest plots were generated, and interaction *p*-values were calculated to assess whether significant interactions existed between different subgroups. Two-tailed *p*-values < 0.05 were considered statistically significant.

## 3 Results

**Figure 1:**
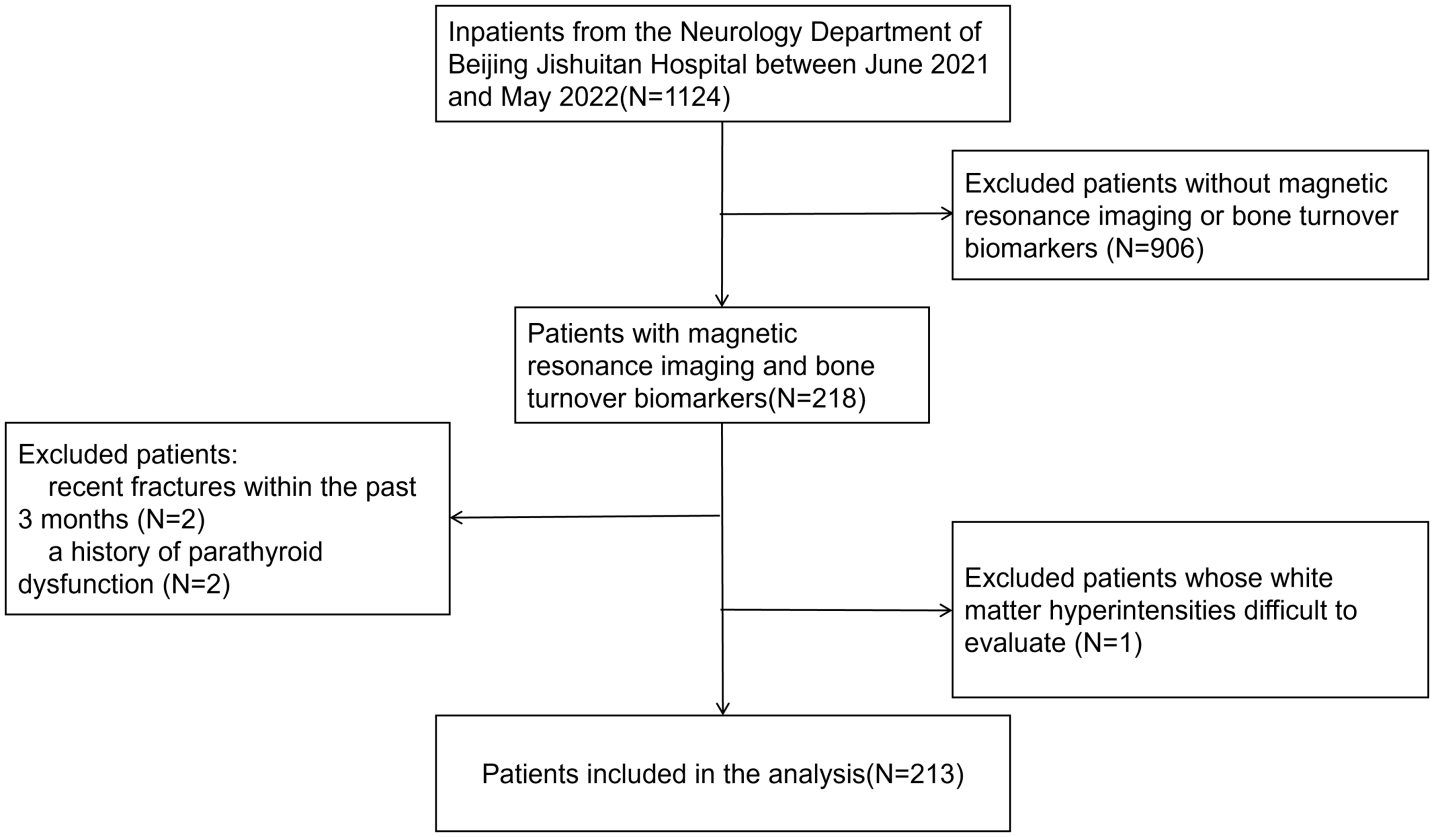
Flowchart of patient inclusion. A presentation of the selection process for the final study population based on the inclusion and exclusion criteria.

A total of 213 patients were included in the study (Figure 1), with a mean age of 69.9 ± 11.6 years. One hundred thirty-nine patients were classified as having mild WMH (ARWMC≤10), while 74 patients had severe WMH (ARWMC>10). Furthermore, 110 patients were classified into the mild PWMH group (Fazekas score 0–1), while 103 patients were in the severe PWMH group (Fazekas score 2–3). Similarly, 110 patients were classified into the mild DWMH group (Fazekas score 0–1), while 103 patients were in the severe DWMH group (Fazekas score 2–3) (Table 1).

**Table 1:** Baseline characteristics of patients.

Characteristic	Total (*n* = 213)	ARWMC^a^			PWMH^b^			DWMH^c^		
		Mild (*n* = 139)	Severe (*n* = 74)	*p*-value	Mild (*n* = 110)	Severe (*n* = 103)	*p*-value	Mild (*n* = 110)	Severe (*n* = 103)	*p*-value
Age, years, Mean ± SD	69.9 ± 11.6	67.9 ± 12.0	73.6 ± 9.7	<0.001	65.9 ± 12.2	74.1 ± 9.1	<0.001	67.5 ± 12.6	72.4 ± 9.8	0.002
Female, *n* (%)	97 (45.5)	63 (45.3)	34 (45.9)	0.931	53 (48.2)	44 (42.7)	0.424	50 (45.5)	47 (45.6)	0.979
Hypertension, *n* (%)	161 (75.6)	100 (71.9)	61 (82.4)	0.09	76 (69.1)	85 (82.5)	0.023	75 (68.2)	86 (83.5)	0.009
DM, *n* (%)	90 (42.3)	56 (40.3)	34 (45.9)	0.426	40 (36.4)	50 (48.5)	0.072	45 (40.9)	45 (43.7)	0.681
Cerebral infarction, *n* (%)	60 (28.2)	32 (23)	28 (37.8)	0.022	22 (20)	38 (36.9)	0.006	23 (20.9)	37 (35.9)	0.015
Smoking, *n* (%)	85 (39.9)	53 (38.1)	32 (43.2)	0.468	43 (39.1)	42 (40.8)	0.802	44 (40)	41 (39.8)	0.977
Obesity, *n* (%)	32 (15.6)	21 (15.4)	11 (15.9)	0.926	16 (14.7)	16 (16.7)	0.696	19 (17.8)	13 (13.3)	0.376
LDL, mmol/l, Mean ± SD	2.50 ± 0.81	2.57 ± 0.78	2.36 ± 0.85	0.077	2.52 ± 0.73	2.48 ± 0.89	0.759	2.56 ± 0.75	2.43 ± 0.87	0.252
HCY, μmol/l, Mean ± SD	18.55 ± 20.69	17.84 ± 23.05	19.88 ± 15.38	0.499	18.69 ± 25.86	18.40 ± 13.20	0.919	18.55 ± 25.56	18.55 ± 13.65	1.000
Scr, μmol/l, Mean ± SD	68.26 ± 18.39	66.28 ± 17.11	71.91 ± 20.18	0.034	66.20 ± 16.16	70.41 ± 20.33	0.097	66.83 ± 17.67	69.75 ± 19.09	0.251
Ca (adjusted), mmol/l, Mean ± SD	2.26 ± 0.09	2.27 ± 0.09	2.25 ± 0.09	0.060	2.27 ± 0.09	2.26 ± 0.09	0.389	2.27 ± 0.09	2.26 ± 0.09	0.498
SP, mmol/l, Mean ± SD	1.04 ± 0.23	1.06 ± 0.24	1.00 ± 0.22	0.081	1.07 ± 0.25	1.02 ± 0.21	0.146	1.07 ± 0.25	1.02 ± 0.21	0.123
tP1NP, ng/ml, Mean ± SD	51.70 ± 24.51	50.99 ± 26.52	53.04 ± 20.32	0.562	51.56 ± 28.38	51.85 ± 19.70	0.932	51.13 ± 28.06	52.31 ± 20.18	0.725
β-CTX, ng/ml, Mean ± SD	0.61 ± 0.31	0.56 ± 0.30	0.70 ± 0.32	0.002	0.55 ± 0.29	0.67 ± 0.32	0.008	0.56 ± 0.32	0.65 ± 0.30	0.034
OC, ng/ml, Mean ± SD	16.11 ± 8.11	15.74 ± 8.58	16.80 ± 7.15	0.366	15.91 ± 8.88	16.32 ± 7.24	0.716	15.56 ± 8.96	16.70 ± 7.10	0.305
PTH, pg./ml, Mean ± SD	44.69 ± 18.14	41.68 ± 18.18	50.34 ± 16.75	<0.001	41.74 ± 17.19	47.84 ± 18.67	0.014	40.40 ± 17.28	49.28 ± 17.99	<0.001
25(OH)VD_3_, ng/ml, Mean ± SD	16.29 ± 7.59	17.06 ± 7.92	14.83 ± 6.75	0.041	17.53 ± 7.78	14.97 ± 7.19	0.014	16.74 ± 8.08	15.81 ± 7.04	0.374

^a^Mild WMH were defined as ARWMC ≤ 10, while severe WMH were defined as ARWMC >10.

^b^Mild PWMH were defined as Fazekas scale 0–1, while severe PWMH were defined as Fazekas scale 2–3.

^c^Mild DWMH were defined as Fazekas scale 0–1, while severe DWMH were defined as Fazekas scale 2–3.

ARWMC, age-related white matter changes; PWMH, periventricular white matter hyperintensities; DWMH, deep white matter hyperintensities; DM, diabetes mellitus; LDL, low-density lipoprotein; HCY, homocysteine; Scr, serum creatinine; Ca, calcium; SP, serum phosphorus; tP1NP, total procollagen type 1N-terminal propeptide; β-CTX, β-carboxy-terminal cross-linked telopeptide of type 1 collagen; OC, N-terminal osteocalcin; PTH, parathyroid hormone; 25 (OH)D_3_, 25-hydroxyvitamin D_3_; SD, standard deviation.

It was shown that β-CTX and PTH levels were significantly higher in patients with severe WMH compared to those with mild WMH (*p* = 0.002 and *p* < 0.001, respectively); they were also higher in the severe PWMH group (*p* = 0.008 and *p* = 0.014, respectively) and in the severe DWMH group (*p* = 0.034 and p < 0.001, respectively). Additionally, it was worth noting that 25 (OH)VD_3_ levels were lower in the severe WMH and severe PWMH groups (*p* = 0.041 and *p* = 0.014, respectively), whereas no significant differences in 25 (OH)VD_3_ levels were observed between the mild and severe DWMH groups (*p* ≥ 0.05). No significant differences were found in tP1NP and OC levels among the different WMH severity groups (*p* ≥ 0.05) (Table 1).

Then, tP1NP, β-CTX, OC and PTH levels were included in the logistic regression model. In unadjusted models, compared with the mild groups, higher β-CTX was associated with severe WMH (OR: 4.23, 95% CI: 1.66–10.76; *p* = 0.003), severe PWMH (OR: 3.40, 95% CI: 1.36–8.53; *p* = 0.009), and severe DWMH (OR: 2.62, 95% CI: 1.06–6.43; *p* = 0.036). Higher PTH showed similar associations: severe WMH (OR: 1.03, 95% CI: 1.01–1.04; *p* = 0.002), severe PWMH (OR: 1.02, 95% CI: 1.00–1.04; *p* = 0.016), and severe DWMH (OR: 1.03, 95% CI: 1.01–1.05; *p* < 0.001). No significant differences were observed in tP1NP and OC levels between the two groups (all *p*-values ≥0.05). After adjusting for confounding factors, elevated β-CTX levels remained significantly associated with severe WMH (OR: 4.44, 95% CI: 1.33–14.81; *p* = 0.015) and severe PWMH (OR: 6.37, 95% CI: 1.80–22.47; *p* = 0.004), while increased PTH levels were significantly associated with severe DWMH (OR: 1.02, 95% CI: 1.01–1.05; *p* = 0.015) (Table 2).

**Table 2:** Relationship between bone turnover markers and white matter hyperintensities based on different classification.

	Unadjusted model		Model 1		Model 2		Model 3	
	OR (95% CI)	*p*-value	OR (95% CI)	*p*-value	OR (95% CI)	*p*-value	OR (95% CI)	*p*-value
ARWMC^a^								
tP1NP	1.00 (0.99 ~ 1.01)	0.561	/	/	/	/	/	/
β-CTX	4.23 (1.66 ~ 10.76)	0.003	3.69 (1.23 ~ 11.12)	0.020	4.25 (1.33 ~ 13.60)	0.015	4.44 (1.33 ~ 14.81)	0.015
OC	1.02 (0.98 ~ 1.05)	0.368	/	/	/	/	/	/
PTH	1.03 (1.01 ~ 1.04)	0.002	1.02 (1.00 ~ 1.03)	0.095	1.02 (1.00 ~ 1.04)	0.073	1.01 (0.99 ~ 1.03)	0.152
PWMH^b^								
tP1NP	1.00 (0.99 ~ 1.01)	0.932	/	/	/	/	/	/
β-CTX	3.40 (1.36 ~ 8.53)	0.009	5.02 (1.57 ~ 16.07)	0.007	6.47 (1.87 ~ 22.41)	0.003	6.37 (1.80 ~ 22.47)	0.004
OC	1.01 (0.97 ~ 1.04)	0.715	/	/	/	/	/	/
PTH	1.02 (1.00 ~ 1.04)	0.016	1.01 (0.99 ~ 1.03)	0.525	1.01 (0.99 ~ 1.03)	0.352	1.01 (0.99 ~ 1.03)	0.488
DWMH^c^								
tP1NP	1.00 (0.99 ~ 1.01)	0.724	/	/	/	/	/	/
β-CTX	2.62 (1.06 ~ 6.43)	0.036	1.70 (0.60 ~ 4.84)	0.320	1.79 (0.59 ~ 5.40)	0.304	1.58 (0.51 ~ 4.89)	0.430
OC	1.02 (0.98 ~ 1.05)	0.307	/	/	/	/	/	/
PTH	1.03 (1.01 ~ 1.05)	<0.001	1.03 (1.01 ~ 1.04)	0.009	1.03 (1.01 ~ 1.05)	0.009	1.02 (1.01 ~ 1.05)	0.015

^a, b, c^With mild as references.

Model 1, adjusted for age, sex, and bone mass index.

Model 2, adjusted for Model 1 + hypertension, diabetes, and cerebral infarction.

Model 3, adjusted for Model 2 + serum creatinine, adjusted calcium, and serum phosphorus.

OR, odds ratio; CI, confidence interval; ARWMC, age-related white matter changes; PWMH, periventricular white matter hyperintensities; DWMH, deep white matter hyperintensities; tP1NP, total procollagen type 1N-terminal propeptide; β-CTX, β-carboxy-terminal cross-linked telopeptide of type 1 collagen; OC, N-terminal osteocalcin; PTH, parathyroid hormone.

**Figure 2:**
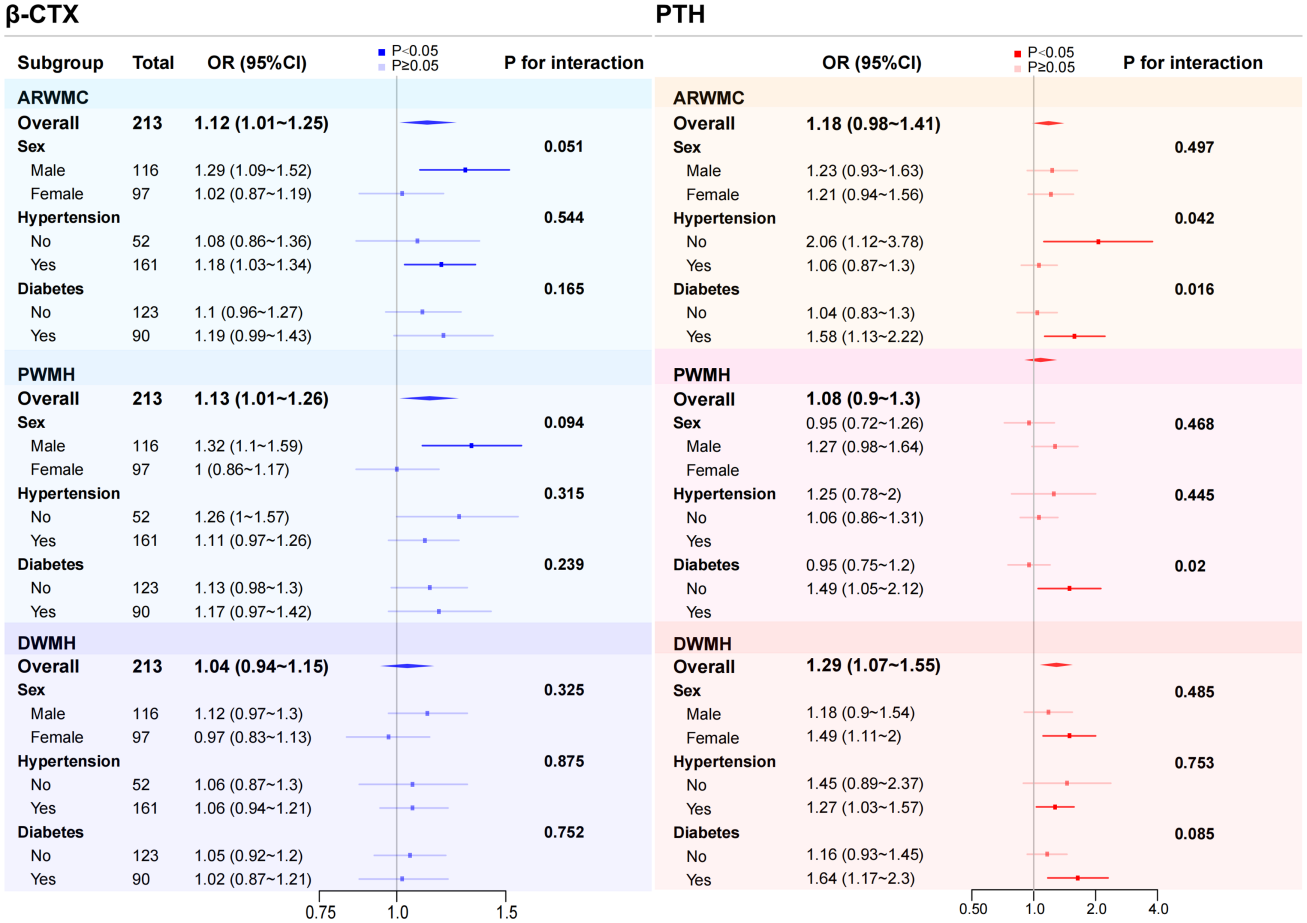
Subgroup analysis and corresponding forest plot of the association between β-CTX and PTH with WMH of varying severity. Subgroup analyses were conducted based on sex, hypertension, and diabetes to examine the association between β-CTX and PTH with different severity of WMH across various subgroups and to explore potential interaction effects. To improve clarity in the presentation, β-CTX levels were multiplied by 10, and PTH levels were divided by 10. This adjustment does not affect the overall trend of the results or the statistical significance (*p*-values). ARWMC, age-related white matter changes; PWMH, periventricular white matter hyperintensities; DWMH, deep white matter hyperintensities; β-CTX, β-carboxy-terminal cross-linked telopeptide of type 1 collagen; PTH, parathyroid hormone; OR, odds ratio; CI, confidence interval.

Subgroup analysis was performed finally. Interaction analysis showed that no significant interaction was found between β-CTX and sex, hypertension or DM status (all *p*-values for interaction ≥ 0.05), suggesting that the relationship may be generally consistent across populations. In contrast, in individuals without hypertension, PTH levels were significantly associated with the severity of WMH (*p* < 0.05), while the association between PTH and PWMH as well as DWMH severity did not differ significantly in the hypertension subgroup (*p* ≥ 0.05). Additionally, in patients with DM, PTH levels were significantly associated with the severity of WMH, PWMH and DWMH (*p* < 0.05), and the associations between PTH and both WMH and PWMH severity differed significantly between diabetic and non-diabetic subgroups (the interaction *p*-values were 0.016 and 0.02, respectively) (Figure 2).

## 4 Discussion

This study is a single-center retrospective study primarily focusing on the correlation between different types of BTMs and PTH with the severity of WMH as assessed by the ARWMC scale and Fazekas score. We found that β-CTX levels were independently and positively associated with the severity of WMH and PWMH, while PTH levels were independently and positively associated with the severity of DWMH.

β-CTX is a degradation product of type 1 collagen formed through matrix metalloproteinases. As a marker of bone resorption, *β*-CTX is released into the bloodstream during osteoclastic degradation of the bone matrix. Elevated β-CTX levels are frequently observed in osteoporosis. In this study, we found that higher β-CTX levels were independently associated with severe WMH and PWMH. Moreover, although the correlation between β-CTX levels and the severity of DWMH was no longer significant after adjusting for confounding factors, the univariate analysis results still suggested a potential trend of association.

Previous studies have demonstrated that patients with reduced BMD were at significantly higher risk of developing cardiovascular and cerebrovascular diseases, independent of traditional vascular risk factors such as age, hypertension, diabetes and smoking (9). Higher levels of CTX have been found in patients with thickening of the carotid intima-media, and these levels are positively related with brachial-ankle pulse wave velocity (11), which are supportive of our results. Several mechanisms may explain the link. First, β-CTX is a circulating fragment released during type 1 collagen breakdown. An elevated β-CTX level therefore signals accelerated degradation of type 1 collagen, a major extracellular-matrix (ECM) component of vessel walls and basement membrane (19). Cerebral small arteriolar remodeling is a hallmark pathological feature of arteriosclerotic CSVD, characterized by lumen narrowing and abnormal ECM accumulation in the vascular wall (20). Deposition of type 1 collagen around cerebral micro-vessels contributes to ECM thickening and fibrosis, leading to reduced vascular elasticity and increased stiffness, as well as disruption of the blood–brain barrier (21, 22). The elevation of β-CTX levels in CSVD may thus also be related to ECM metabolic disturbances in vascular walls. Second, altered mineral metabolism and the cross talk between bone and cardiovascular system play an important role in atherosclerosis or vascular calcification (23). Osteoporosis and vascular calcification may arise through two routes. In the active route, vascular cells switch to an osteoblast-like state, secrete ECM, and mineralize it. In the passive route, excess calcium and phosphate precipitate in damaged vessel walls, where macrophages fail to clear the deposits (24). Both processes may occur together, so higher bone resorption and mineral-balance disorders are linked to vascular calcification. In this study, serum calcium and phosphate levels did not show significant differences among groups with varying severity of WMH, but this could not completely rule out the potential pathophysiological role. The last but not the least, studies show that pro-inflammatory molecules such as interleukin-1, interleukin-6, and tumor necrosis factor-*α* both stimulate osteoclast activity, which could raise β-CTX levels, and promote atherosclerosis by triggering endothelial dysfunction and plaque inflammation (25, 26). The receptor activator of nuclear factor-κB/receptor activator of nuclear factor-κB ligand/osteoprotegerin (RANK/RANKL/OPG) system, part of the tumor necrosis factor superfamily, regulates osteoclast activity and is expressed in vascular endothelial and inflammatory cells. Up-regulation of RANKL not only increase bone resorption but also raises matrix metalloproteinases activity in vascular smooth muscle cells (VSMCs), accelerating matrix degradation and remodeling of the vessel wall (25). These findings imply that bone and cerebrovascular disease may share inflammatory pathways, though this still needs confirmation.

PTH is a key hormone regulating bone metabolism. Our study found that PTH levels were independently related with the severity of DWMH. PTH levels also showed a trend to be correlated with the severity of WMH and PWMH, though this association did not remain significant after adjusting for confounding factors. Previous clinical studies on the role of PTH on cerebrovascular diseases have shown mixed results. A study from the Uppsala Longitudinal Study of Adult Men (ULSAM) found a significant link between PTH levels and vascular dementia (14), though the sample size was small. Another analysis from the Prospective Investigation of the Vasculature in Uppsala Seniors (PIVUS) showed a significant correlation between PTH levels and WMH severity (14). A study from the Atherosclerosis Risk in Communities (ARIC) cohort found that higher PTH levels were linked to more severe WMH, but this association disappeared after adjustment for traditional risk factors such as hypertension and diabetes (27). As noted above, increased PTH is related to elevated blood pressure, and parathyroidectomy has been reported to improve both blood pressure and glycemic control (28). These observations raise the possibility that hypertension, and perhaps hyperglycemia, mediates any contribution of PTH to CSVD. In addition, in individuals with primary hyperparathyroidism, the mean carotid intima-media thickness is significantly increased, and higher PTH levels are associated with worse carotid stiffness, reduced carotid strain and decreased distensibility (29). These studies all suggest a probable correlation between PTH and CSVD.

There were several potential mechanisms. First, Chronic PTH excess drives endothelial cells to generate excess mitochondrial reactive oxygen species, causing endothelium-dependent vasodilatory dysfunction and abnormal vascular remodeling, which promote the development of CSVD (30). Second, PTH may activate several signaling pathways to stimulate the renin–angiotensin system by binding to PTH/PTH-related peptide (PTHrP) receptors, thus triggering renin release and aldosterone secretion, resulting in rise of blood pressure and volume load (31). Third, because PTH is a key regulator of calcium–phosphate balance, a sustained rise in its level can disrupt mineral homeostasis, promote vascular calcification, and stiffen arteries (24). PTH has been shown to increase collagen synthesis and remodeling in VSMCs (29, 32), potentially mediating vascular stiffening and reduced elasticity, thereby aggravating CSVD. Therefore, it is speculated that PTH may contribute to the occurrence and progression of WMH by impairing endothelial function, raising blood pressure, disturbing calcium-phosphate balance, and promoting vascular remodeling.

In addition, in our study, subgroup analysis revealed that PTH levels were significantly associated with the severity of WMH in individuals with DM. This association was not observed in non-diabetic individuals. Chronic hyperglycemia can promote WMH by generating oxidative stress, impairing endothelium-dependent relaxation, disrupting endothelial tight junctions, triggering inflammation, and accelerating atherosclerotic vascular remodeling (33, 34). We hypothesize that on this “more vulnerable” white-matter background, the additional vascular stress caused by excess PTH may be easier to detect as a higher WMH burden. Diabetic kidney disease compounds the problem: even in early stages (chronic kidney disease 2–4), mineral-balance disturbances appear (35), which might magnify the PTH–WMH link. Notably, PTH itself has been reported to be associated with incident diabetes, although the mechanism remains unclear (36). This finding suggests that future research should further explore the role of PTH in WMH among patients with DM, and consider whether modulating PTH levels could serve as a potential intervention for diabetes-related WMH.

This study has several limitations. First, as a retrospective study, it lacked explicit documentation in medical records about whether patients had a history of osteoporosis, and BMD was not measured. As a result, the analysis focused solely on the relationship between BTMs and WMH, without investigating a direct link between osteoporosis and WMH. Second, the sample size was small. While the findings indicate a potential association between CTX, PTH and the severity of WMH, causality cannot be established. Further validation through larger-scale studies is still needed. Finally, because the archived scans did not record magnet strength, we could not adjust for MRI field strength (1.5T or 3.0T) or perform subgroup analyses, which may have influenced the WMH scores.

As the prevalence of CSVD continues to rise and effective treatments remain limited, this study offers early evidence of a possible connection between bone metabolism and vascular health. The findings suggest that β-CTX and PTH may have biological relevance to CSVD. In practice, β-CTX is usually tracked in patients with osteoporosis. If the marker rises sharply, especially in older or hypertensive patients, or in those developing cognitive or gait problems, a brain MRI could be considered to identify potential CSVD. Likewise, patients with primary or secondary hyperparathyroidism might benefit from CSVD screening, guiding more proactive management. The reverse also applies. In patients with a heavy WMH burden, checking parathyroid function may be sensible, and those at high risk of falls, which is a common issue in CSVD, could be screened for osteoporosis. While these suggestions are hypothesis-generating, larger studies are needed to clarify both the associations and any causal links. Besides, this work is an exploratory correlation study; large-scale cohorts are needed to assess the value of β-CTX and PTH as potential biomarkers for CSVD, especially WMH. Notably, our subgroup analysis showed a stronger PTH–WMH link in patients with diabetes than in those without, suggesting that future studies could examine this relationship in larger diabetic samples. Mechanistic research is also required to clarify how β-CTX and PTH drive disease processes and to explore their potential as therapeutic targets.

## 5 Conclusion

Independent associations were observed between β-CTX and WMH/PWMH severity and between PTH and DWMH severity. This study provides early evidence linking osteo-metabolic markers (β-CTX and PTH) with CSVD burden, suggesting a plausible bone–cerebrovascular axis. Prospective cohort and interventional studies are needed to evaluate their potential as biomarkers and therapeutic targets, and elucidate the underlying mechanisms.

## Data Availability

The raw data supporting the conclusions of this article will be made available by the authors, without undue reservation.

## References

[1] Markus HS de Leeuw FE. Cerebral small vessel disease: recent advances and future directions. Int J Stroke. (2022) 18:4–14. doi: 10.1177/17474930221144911, PMID: 36575578 PMC9806465

[2] Lam BYK Cai Y Akinyemi R Biessels GJ van den Brink H Chen C. The burden of cerebral small vessel disease in low-and middle-income countries – a systematic review and meta-analysis. Int J Stroke. (2023) 18:15–27. doi: 10.1177/17474930221137019 36282189

[3] Das AS Regenhardt RW Vernooij MW Blacker D Charidimou A Viswanathan A. Asymptomatic cerebral small vessel disease: insights from population-based studies. J Stroke. (2019) 21:121–38. doi: 10.5853/jos.2018.03608, PMID: 30991799 PMC6549070

[4] de Havenon A Smith EE Sharma R Falcone GJ Bangad A Prabhakaran S. Improvement in the prediction of cerebrovascular events with white matter Hyperintensity. J Am Heart Assoc. (2023) 12:e029374. doi: 10.1161/JAHA.123.029374, PMID: 37345754 PMC10356061

[5] Karsenty G Olson EN. Bone and muscle endocrine functions: unexpected paradigms of inter-organ communication. Cell. (2016) 164:1248–56. doi: 10.1016/j.cell.2016.02.043, PMID: 26967290 PMC4797632

[6] Liu T Wu H Li J Zhu C Wei J. Unraveling the bone-brain Axis: a new frontier in Parkinson's disease research. Int J Mol Sci. (2024) 25:12842. doi: 10.3390/ijms252312842, PMID: 39684552 PMC11641043

[7] Zhang F Zhang W. Research progress in Alzheimer's disease and bone-brain axis. Ageing Res Rev. (2024) 98:102341. doi: 10.1016/j.arr.2024.102341, PMID: 38759893

[8] Szulc P. Bone turnover: biology and assessment tools. Best Pract Res Clin Endocrinol Metab. (2018) 32:725–38. doi: 10.1016/j.beem.2018.05.003, PMID: 30449551

[9] Anagnostis P Karagiannis A Kakafika AI Tziomalos K Athyros VG Mikhailidis DP. Atherosclerosis and osteoporosis: age-dependent degenerative processes or related entities? Osteoporos Int. (2008) 20:197–207. doi: 10.1007/s00198-008-0648-5 18509713

[10] Kim JM Park KY Kim HR Ahn HY Pantoni L Park MS. Association of bone mineral density to cerebral small vessel disease burden. Neurology. (2021) 96:e1290–300. doi: 10.1212/WNL.0000000000011526, PMID: 33431517

[11] Kondo T Endo I Aihara K Onishi Y Dong B Ohguro Y. Serum carboxy-terminal telopeptide of type I collagen levels are associated with carotid atherosclerosis in patients with cardiovascular risk factors. Endocr J. (2016) 63:397–404. doi: 10.1507/endocrj.EJ15-0589, PMID: 26877258

[12] Altay H Altın C Coner A Muderrisoglu H Giray S. Parathyroid hormone and ischemic cerebrovascular event. Endocr Metab Immune Disord Drug Targets. (2019) 19:1134–40. doi: 10.2174/1871530319666190215150410, PMID: 30806331

[13] Chung PW Park KY Kim JM Shin DW Park MS Chung YJ. 25-Hydroxyvitamin D status is associated with chronic cerebral small vessel disease. Stroke. (2015) 46:248–51. doi: 10.1161/STROKEAHA.114.007706, PMID: 25424481

[14] Hagström E Kilander L Nylander R Larsson E-M Michaëlsson K Melhus H. Plasma parathyroid hormone is associated with vascular dementia and cerebral Hyperintensities in two community-based cohorts. J Clin Endocrinol Metabol. (2014) 99:4181–9. doi: 10.1210/jc.2014-1736, PMID: 25140397

[15] Broulik PD Broulikova A Adamek S Libansky P Tvrdon J Broulikova K. Improvement of hypertension after parathyroidectomy of patients suffering from primary hyperparathyroidism. Int J Endocrinol. (2011) 2011:309068. doi: 10.1155/2011/309068, PMID: 21403888 PMC3043284

[16] Nilsson IL Aberg J Rastad J Lind L. Endothelial vasodilatory dysfunction in primary hyperparathyroidism is reversed after parathyroidectomy. Surgery. (1999) 126:1049–55. doi: 10.1067/msy.2099.101422, PMID: 10598187

[17] Zeng Q Li N Pan XF Chen L Pan A. Clinical management and treatment of obesity in China. Lancet Diabetes Endocrinol. (2021) 9:393–405. doi: 10.1016/S2213-8587(21)00047-4, PMID: 34022157

[18] He X Lin B Tao T Chen Q Wang J Jin J. Higher serum albumin-corrected calcium levels are associated with revascularization and poor outcome after mechanical thrombectomy. BMC Neurol. (2022) 22:330. doi: 10.1186/s12883-022-02856-2, PMID: 36056314 PMC9438214

[19] Selvaraj V Sekaran S Dhanasekaran A Warrier S. Type 1 collagen: synthesis, structure and key functions in bone mineralization. Differentiation. (2024) 136:100757. doi: 10.1016/j.diff.2024.100757, PMID: 38437764

[20] Liu N Tang J Xue Y Mok V Zhang M Ren X. EP3 receptor deficiency improves vascular remodeling and cognitive impairment in cerebral small vessel disease. Aging Dis. (2022) 13:313–28. doi: 10.14336/AD.2021.0627, PMID: 35111376 PMC8782563

[21] Özkan E Çetin-Taş Y Şekerdağ E Kızılırmak AB Taş A Yıldız E. Blood–brain barrier leakage and perivascular collagen accumulation precede microvessel rarefaction and memory impairment in a chronic hypertension animal model. Metab Brain Dis. (2021) 36:2553–66. doi: 10.1007/s11011-021-00767-8, PMID: 34118020

[22] I C G RF. Neurovascular and cognitive dysfunction in hypertension. Circ Res. (2019) 124:1025–44. doi: 10.1161/CIRCRESAHA.118.313260 30920929 PMC6527115

[23] Goettsch C Iwata H Aikawa E. Parathyroid hormone: critical bridge between bone metabolism and cardiovascular disease. Arterioscler Thromb Vasc Biol. (2014) 34:1333–5. doi: 10.1161/ATVBAHA.114.303637, PMID: 24951650 PMC4094346

[24] Hofbauer LC Brueck CC Shanahan CM Schoppet M Dobnig H. Vascular calcification and osteoporosis--from clinical observation towards molecular understanding. Osteoporos Int. (2007) 18:251–9. doi: 10.1007/s00198-006-0282-z, PMID: 17151836

[25] Zupan J Jeras M Marc J. Osteoimmunology and the influence of pro-inflammatory cytokines on osteoclasts. Biochem Med. (2013) 23:43–63. doi: 10.11613/BM.2013.007, PMID: 23457765 PMC3900089

[26] Rochette L Meloux A Rigal E Zeller M Cottin Y Vergely C. The role of Osteoprotegerin and its ligands in vascular function. Int J Mol Sci. (2019) 20:705. doi: 10.3390/ijms20030705, PMID: 30736365 PMC6387017

[27] Korada SKC Zhao D Gottesman RF Guallar E Lutsey PL Alonso A. Parathyroid hormone and subclinical cerebrovascular disease: the atherosclerosis risk in communities brain magnetic resonance imaging study. J Stroke Cerebrovasc Dis. (2016) 25:883–93. doi: 10.1016/j.jstrokecerebrovasdis.2015.12.029, PMID: 26825350 PMC4799747

[28] Kumar A Singh S. Parathyroidectomy ameliorates glucose and blood pressure control in a patient with primary hyperparathyroidism, type 2 diabetes, and hypertension. Clin Med Insights Endocrinol Diabetes. (2015) 8:63–6. doi: 10.4137/CMED.S31292, PMID: 26380561 PMC4559184

[29] Walker MD Fleischer J Rundek T McMahon DJ Homma S Sacco R. Carotid vascular abnormalities in primary hyperparathyroidism. J Clin Endocrinol Metab. (2009) 94:3849–56. doi: 10.1210/jc.2009-1086, PMID: 19755478 PMC2758727

[30] Gambardella J De Rosa M Sorriento D Prevete N Fiordelisi A Ciccarelli M. Parathyroid hormone causes endothelial dysfunction by inducing mitochondrial ROS and Specific oxidative signal transduction modifications. Oxidative Med Cell Longev. (2018) 2018:9582319. doi: 10.1155/2018/9582319, PMID: 30662585 PMC6313989

[31] Zheng MH Li FX Xu F Lin X Wang Y Xu QS. The interplay between the renin-angiotensin-aldosterone system and parathyroid hormone. Front Endocrinol. (2020) 11:539. doi: 10.3389/fendo.2020.00539, PMID: 32973674 PMC7468498

[32] Perkovic V Hewitson TD Kelynack KJ Martic M Tait MG Becker GJ. Parathyroid hormone has a Prosclerotic effect on vascular smooth muscle cells. Kidney Blood Press Res. (2003) 26:27–33. doi: 10.1159/000069761, PMID: 12697974

[33] Sun J Xu B Zhang X He Z Liu Z Liu R. The mechanisms of type 2 diabetes-related white matter intensities: a review. Front Public Health. (2020) 8:498056. doi: 10.3389/fpubh.2020.498056, PMID: 33282807 PMC7705244

[34] An Y Xu BT Wan SR Ma XM Long Y Xu Y. The role of oxidative stress in diabetes mellitus-induced vascular endothelial dysfunction. Cardiovasc Diabetol. (2023) 22:237. doi: 10.1186/s12933-023-01965-7, PMID: 37660030 PMC10475205

[35] Wahl P Xie H Scialla J Anderson CA Bellovich K Brecklin C. Earlier onset and greater severity of disordered mineral metabolism in diabetic patients with chronic kidney disease. Diabetes Care. (2012) 35:994–1001. doi: 10.2337/dc11-2235, PMID: 22446176 PMC3329844

[36] Williams A Zhao S Brock G Kline D Echouffo-Tcheugui JB Effoe VS. Vitamin D, parathyroid hormone, glucose metabolism and incident diabetes in the multiethnic study of atherosclerosis. BMJ Open Diabetes Res Care. (2022) 10:e002931. doi: 10.1136/bmjdrc-2022-002931, PMID: 36162866 PMC9516211

